# Expression of Neurofilament Subunits at Neocortical Glutamatergic and GABAergic Synapses

**DOI:** 10.3389/fnana.2018.00074

**Published:** 2018-09-11

**Authors:** Luca Bragina, Fiorenzo Conti

**Affiliations:** ^1^Section of Neuroscience and Cell Biology, Department of Experimental and Clinical Medicine, Università Politecnica delle Marche, Ancona, Italy; ^2^Center for Neurobiology of Aging, IRCCS INRCA, Ancona, Italy

**Keywords:** glutamate, GABA, neurofilaments, synapses, cerebral cortex

## Abstract

Neurofilaments (NFs) are neuron-specific heteropolymers that have long been considered as structural proteins. However, it has recently been documented that they may play a functional role at synapses. Indeed, the four NF subunits—NFL, NFM, NFH and α-internexin—are integral components of synapses in the striatum and hippocampus, since their elimination disrupts synaptic plasticity and impairs social memory, an observation that might have important implications for some neuropsychiatric diseases. Here, we studied NFs localization in VGLUT1-, VGLUT2-, VGAT-, PSD-95- and gephyrin-positive (+) puncta, and in glutamatergic and GABAergic synapses in the cerebral cortex of adult rats. Synapses were identified by pre- and postsynaptic markers: glutamatergic synapses by VGLUT1+ or VGLUT2+ puncta contacting PSD-95+ puncta; and GABAergic synapses by VGAT+ puncta contacting gephyrin+ puncta. In VGLUT1 glutamatergic synapses NF showed a greater expression in the compartment labeled by postsynaptic markers (20%–30%) than in those labeled by presynaptic markers (10%–20%), whereas in GABAergic synapses a similar expression was detected in both compartments (20%–30%). Moreover, NF expression was higher in the GABAergic (20%–30%) than in the glutamatergic (10%–15%) compartments labeled by presynaptic markers. Finally, a higher colocalization of VGLUT1+, VGLUT2+ and VGAT+ puncta with NFs was seen when presynaptic puncta contacted elements labeled by postsynaptic markers. These findings show that the four NF subunits are expressed at some neocortical synapses, and contribute to glutamatergic and GABAergic synapse heterogeneity.

## Introduction

Glutamatergic and GABAergic synapses are characterized by a broad variability of response that depends on factors acting presynaptically, at the cleft, or postsynaptically (Conti and Weinberg, 1999; Cherubini and Conti, 2001). The heterogeneous expression of presynaptic proteins is associated with variability in neurotransmitter release (Staple et al., 2000). In the postsynaptic compartment, the expression of neurotransmitter receptors shows extensive structural diversification due to the assembly of different subunits (Mackler and Eberwine, 1993; Huntley et al., 1994; Fritschy and Mohler, 1995; Craig and Boudin, 2001; Paoletti, 2011). Many other molecules, including scaffold and cytoskeletal proteins, contribute to synaptic heterogeneity (van Rossum and Hanisch, 1999; Craig and Boudin, 2001).

Following the demonstration that more than 2,000 genes are differentially expressed in glutamatergic and GABAergic neurons (Sugino et al., 2006), we investigated whether the release machineries of such neurons could be differentiated based on the proteins they express. We found that the expression pattern of several presynaptic proteins involved in transmitter release in cerebral cortex (including synapsins, synaptophysins, synaptosomal-associated proteins, synaptogyrins, synaptobrevin/vesicle-associated membrane proteins, syntaxins, synaptotagmins, synaptic vesicle proteins and Rab3) varies both between glutamatergic and GABAergic terminals and between VGLUT1- and VGLUT2-positive (+) glutamatergic terminals (Bragina et al., 2007, 2010, 2012). We also demonstrated that metabotropic glutamate receptors and GABA_B_ also show heterogeneous expression in glutamatergic and GABAergic presynaptic terminals (Bragina et al., 2015).

Neurofilaments (NFs) are heteropolymers that include four known subunits, NFL, NFM, NFH and α-internexin (INT). They have long been considered as structural proteins required for the radial growth of axons and to support the dendrites of large motor neurons. Recently, however, they have been shown to play a functional role at synapses, since a study by Yuan et al. (2015) found that their elimination disrupted synaptic plasticity and impaired social memory, with potentially important implications for some neuropsychiatric diseases. In the striatum and hippocampus, all NF subunits were expressed both at presynaptic and postsynaptic sites, although their expression was greater in postsynaptic regions. NF assemblies isolated from synapses were different from those found in other portions of the neurons, and showed a higher proportion of INT and NFH phosphorylation states and a lower proportion of NFM phosphorylation states (Yuan et al., 2015).

The presence of NF subunits at synaptic sites raises the possibility that they contribute to the synaptic heterogeneity of glutamatergic and GABAergic synapses. To verify this hypothesis, we studied the localization of the four NFs in VGLUT1-, VGLUT2-, VGAT-, PSD-95 and gephyrin (GEPH)+ puncta and in glutamatergic and GABAergic synapses in adult rat cerebral cortex. We report here that all NF subunits are localized at glutamatergic and GABAergic cortical synapses both in compartments labeled by presynaptic markers and in those labeled by postsynaptic markers, and that their expression contributes to the heterogeneity of these synapses.

## Materials and Methods

### Animals and Tissue Preparation

Adult male Sprague-Dawley rats (190–220 g; Charles River, Milano, Italy) were used. Their care and handling was approved by the local animal research ethics committee. All experimental procedures involving animals and their care were carried out in accordance with national regulations (D.L. no. 26, March 14, 2014) and European Communities Council Directive (2010/63/UE) guidelines and were approved by the local authority veterinary services. Animals were kept in a 12 h dark-light cycle and provided with food and water *ad libitum*.

For western blotting, two rats were anesthetized with chloral hydrate (300 mg/kg i.p.) and their brains were rapidly removed. Neocortex homogenization, membrane preparation, protein determination, SDS-PAGE analysis and immunoblotting were as described previously (Bragina et al., 2006). Precast gels (Tris-HCl; BioRad, Hercules, CA, USA) were used at a 4%–20% polyacrylamide concentration for NFH and NFM (10 μg total protein; 2 gels/animal) and at 7.5% for the NFL and α-internexin (10 μg total protein; 2 gels/animal).

For immunocytochemical studies, 12 rats were anesthetized with chloral hydrate (300 mg/kg i.p.) and perfused through the ascending aorta with saline followed by 4% paraformaldehyde in 0.1 M phosphate buffer (PB; pH 7.4). Brains were postfixed for 2 h at 4°C in the same fixative, cut with a Vibratome into 50 μm thick sections, and processed as described previously (Bragina et al., 2010).

### Antibodies

The primary and secondary antibodies used in the study are listed in Table 1. Western blotting was performed to verify antibody specificity; nitrocellulose filters were probed with antibodies against VGLUT1, VGLUT2, VGAT, NFL, NFM, NFH, INT, PSD-95 and GEPH at the dilutions reported in Table 1. After exposure to the appropriate peroxidase-conjugated antibodies, immunoreactive bands were visualized by BioRad Chemidoc and Quantity One software (BioRad, Hemel Hempstead, UK) using the SuperSignal West Pico (Rockford, IL) chemiluminescent substrate.

**Table 1:** Primary and Secondary antibodies.

A. Primary antibodies						
	Host°	Dilution*	Source	Characterization	RRID
VGAT	GP	1:500 (IF)/1:1,000 (WB)	Synaptic System/131004	Mikhaylova et al. (2014); Fekete et al. (2015)	AB_887873
VGLUT1	GP	1:800 (IF)/1:2,000 (WB)	Millipore/AB5905	Melone et al. (2005)	AB_2301751
VGLUT2	GP	1:800 (IF)/1:2,000 (WB)	Millipore/AB5907	Cubelos et al. (2005); Liu et al. (2005)	AB_2301731
PSD-95	M	1:300 (IF)/1:500 (WB)	UC Davis/NeuroMab/75-028 (K28/43)	Kim et al. (1995)	AB_2307331
GEPH	M	1:200 (IF)/1:500 (WB)	Synaptic System/147021	Mikhaylova et al. (2014); Fekete et al. (2015)	AB_1279448
NFL	M	1:500 (IF)/1:1,000 (WB)	SIGMA/N5139 (NR4)	Yuan et al. (2015)	AB_477276
NFL	Rb	1:200 (IF)/1:1,000 (WB)	Millipore/AB9568	Yuan et al. (2012)	AB_570618
NFM	M	1:600 (IF)/1:1,000 (WB)	SIGMA/N5264 (NN18)	Yuan et al. (2015)	AB_477278
NFM	Rb	1:200 (IF)/1:1,000 (WB)	Millipore/AB1987	Yuan et al. (2015)	AB_91201
NFH	M	1:1,000 (IF)/1:1,000 (WB)	SIGMA/N0142 (N52)	Yuan et al. (2015)	AB_477257
NFH	Rb	1:200 (IF)/1:2,000 (WB)	SIGMA/N4142	Yuan et al. (2015)	AB_477272
INT	M	1:500 (IF)/1:1,000 (WB)	Millipore/MAB5224	Yuan et al. (2015)	AB_2127486
INT	Rb	1:1,000 (IF)/1:1,000 (WB)	Millipore/AB5354	Yuan et al. (2015)	AB_91800
**B. Secondary antibodies**				
**Conjugated to**	**Reacting to**°	**Dilution**	**Source: Jackson ImmunoResearch**		**RRID**
Peroxidase	GP	1:4,000	PA/706-036-148		AB_2340448
Peroxidase	M	1:4,000	PA/715-036-151		AB_2340774
Peroxidase	Rb	1:4,000	PA/711-036-152		AB_2340590
Alexa Fluor^®^ 488	GP	1:250	PA/706-546-148		AB_2340473
Alexa Fluor^®^ 488	M	1:250	PA/715-546-150		AB_2340849
Cy^TM^3	Rb	1:250	PA/711-166-152		AB_2313568
Alexa Fluor^®^ 647	GP	1:250	PA/706-606-148		AB_2340477

°GP, guinea pig; M, mouse; Rb; rabbit; *IF, immunofluorescence; WB, western blotting.

### Colocalization Studies

**Figure 1:**
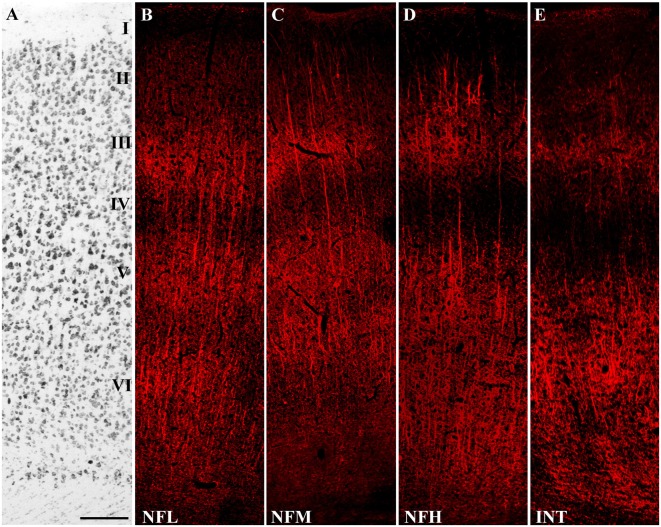
Distribution of Neurofilaments (NFs) in cerebral neocortex. **(A)** Nissl-stained section from the first somatic sensory cortex (SI). **(B–E)** Sections adjacent to that illustrated in **(A)** showing immunoreactivity to NFL, NFM, NFH and INT, in the order. Bar: 100 μm for **(A–E)**.

Sections were incubated for 1 h in normal goat serum (NGS; 10% in PB) and then overnight at room temperature in a solution containing a mixture of the primary antibodies (Table 1). The next day, sections were incubated in 10% NGS for 30 min and then for 90 min in a mixture of the appropriate secondary fluorescent antibodies (Table 1). Double- and triple-labeled sections were examined using a Leica (TCS SP2) confocal laser microscope equipped with an argon and a helium/neon laser. Green, red and blue immunofluorescence were imaged sequentially. Control experiments with single-labeled sections and sections incubated either with two primary and one secondary antibody or with one primary and two secondary antibodies revealed no appreciable fluorochrome bleed-through or antibody cross-reactivity. Images of experimental series were collected from a region of the parietal cortex characterized by a conspicuous layer IV, with intermingled dysgranular regions, densely packed layers II and III, and a relatively cell-free layer Va (e.g., Figure 1). This area corresponds to the first somatic sensory cortex (SI) as also identified in other studies (Woolsey and LeMessurier, 1948; Welker, 1971; Zilles et al., 1980; Donoghue and Wise, 1982). Images were acquired from randomly selected subfields in layers II-VI (at least 4–6/layer; 2–4 sections/animal). Layer I was not sampled because it contains hardly any VGAT+ puncta (Chaudhry et al., 1998; Minelli et al., 2003). Images were acquired using a 60× oil immersion lens (numerical aperture, 1.4; pinhole, 1.0; image size, 512 × 512, pixel size, 0.80 μm) from a plane in which the resolution of all stains was satisfactory, in general 1.3–1.8 μm from the surface. To improve the signal/noise ratio, 10 frames/image were averaged.

**Figure 2:**
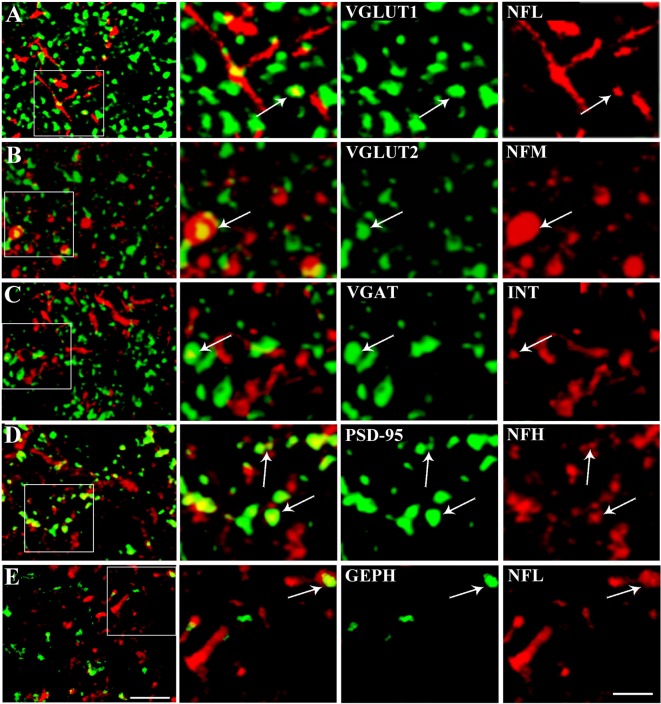
Representative confocal microscopical images showing VGLUT1/NFL (row **A**), VGLUT2/NFM (row **B**), VGAT/INT (row **C**), PSD/NFH (row **D**) and GEPH/NFL colocalization (row **E**). Enlargements show each channel separately, the synaptic marker in green and the NF in red. Puncta were considered double-labeled (arrow) when they virtually overlapped or when a punctum was entirely included in the other. Bar: 5 μm, enlargements, 2 μm.

**Figure 3:**
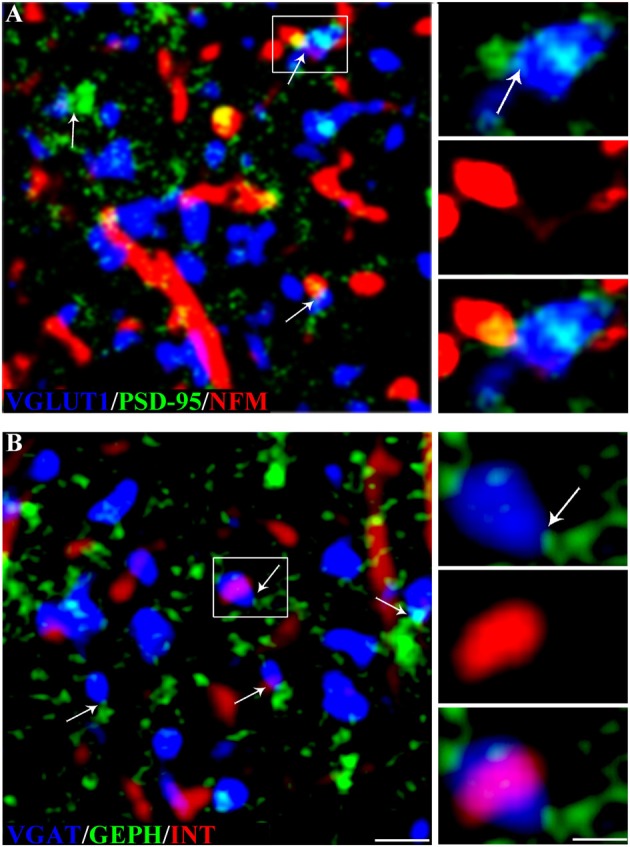
NF expression in glutamatergic and GABAergic synapses in rat cerebral cortex. **(A)** VGLUT1 (blue), PSD-95 (green) and NFM (red) in triple-labeled sections. The arrows indicate glutamatergic synapses (VGLUT1+ puncta contacting PSD-95+ puncta). The image shows a synapse with an NFM+ postsynaptic element; the enlargements show the glutamatergic synapse (top), the NFM+ punctum (middle), and the triple label (bottom). **(B)** VGAT+ (blue), GEPH+ (green) and INT+ (red) puncta in rat triple-labeled sections. The arrows indicate GABAergic synapses (VGAT+ puncta contacting GEPH+ puncta). The image shows a synapse with an INT+ presynaptic element; the enlargements show the GABAergic synapse (top), the INT+ punctum (middle), and the triple label (bottom). Puncta were considered double-labeled when they virtually overlapped or when a punctum was entirely included in the other. Bar: 2 μm, enlargements 1 μm.

Quantitative analysis was performed in ~8,000 randomly selected subfields measuring ~25 × 25 μm from the 512 × 512 pixel images. Images were deconvolved using the Iterative Deconvolve 3D plugin of ImageJ software (v. 1.48, NIH), using the same parameters for all images (Marcotulli et al., 2017). For double-labeled sections, the two channels were first examined separately, to identify and count immunopositive puncta using ImageJ software; the channels were then merged and the number of colocalizing puncta was counted manually. Puncta were considered double-labeled when they showed virtually complete overlap and the morphology of the puncta was coincident, or when a punctum was entirely included in the other (Figure 2; Bragina et al., 2007). The manual count was subsequently confirmed with computerized overlap analysis (object-based analysis), which is included in the ImageJ JACoP toolbox (Bolte and Cordelières, 2006; Bragina et al., 2015), where two objects are considered as colocalizing if the center of mass of one falls within the area of the other (Lachmanovich et al., 2003). About half of all double-labeled sections of each type of colocalization were subjected to object-based analysis. The manual and computerized method obtained comparable results. Triple-labeled sections were evaluated to investigate synapses. Glutamatergic synapses were identified by VGLUT1+ or VGLUT2+ puncta contacting PSD-95+ puncta (Figure 3), whereas GABAergic synapses were identified by VGAT+ puncta contacting GEPH+ puncta (Figure 3; Melone et al., 2005). Finally, colocalization of the four NF subunits in the compartments identified by pre- and post-synaptic markers was assessed with the manual count method used to count double-labeled sections. Rotation of one channel by 90° was used as a specificity control; the results showed that co-localization was always <5%.

### Statistical Analysis

Statistical significance in triple-labeling experiments was evaluated by non-parametric one-way ANOVA with Dunn’s post-test using GraphPad Prism Software (v. 6.0; GraphPad Software, San Diego, CA, USA).

## Results

**Figure 4:**
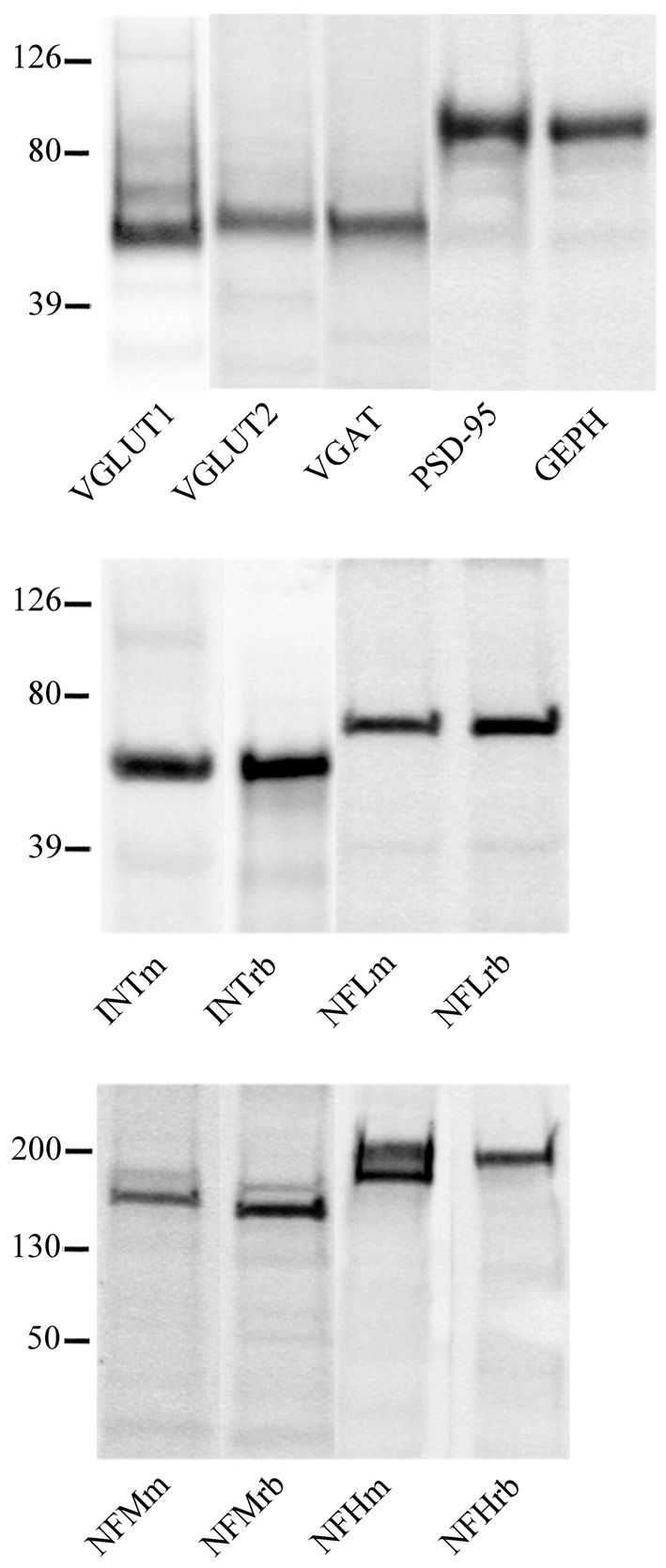
VGLUT1, VGLUT2, VGAT, PSD-95, GEPH, INTm, INTrb, NFLm, NFLrb, NFMm, NFMrb, NFHm and NFHrb antibodies recognized bands of ~55, 60, 57, 95, 93, 58, 58 68, 68, 160, 145, 200 (with phosphorylation site) and 200 kDa, in this order in crude membrane fractions of rat cerebral cortex.

Western blotting studies showed that all antibodies recognized bands with the predicted molecular mass in cortical crude membrane fractions (Figure 4; Prior et al., 1992; Kim et al., 1995; Bellocchio et al., 1998; Chaudhry et al., 1998; Varoqui et al., 2002; Yuan et al., 2015). In immunocytochemical preparations, VGLUT1, VGLUT2, VGAT, PSD-95, GEPH, NFL, NFM, NFH, and INT immunoreactivity were as described in previous studies (Kirsch and Betz, 1993; Suzuki et al., 1997; Bellocchio et al., 1998; Chaudhry et al., 1998; Valtschanoff et al., 1999; Kaneko et al., 2002; Kirkcaldie et al., 2002; Minelli et al., 2003; Alonso-Nanclares et al., 2004; Conti et al., 2005; Figure 1); therefore antibodies were employed to establish whether NFL, NFM, NFH and INT are differentially expressed in VGLUT1+, VGLUT2+, VGAT+, PSD-95+ and GEPH+ puncta and in glutamatergic and GABAergic synapses. The results of these experiments are reported in Tables 2, 3.

**Table 2:** Colocalization of NFL, NFM, NFH and INT in VGLUT1+, VGLUT2+, VGAT+, PSD-95+ and GEPH+ puncta in the cerebral cortex.

	JACoP count		Manual count		NF subunits
	Puncta (no.)	Colocalization (%)	Puncta (no.)	Colocalization (%)	
VGLUT1	2450	(7.11 ± 0.35%)	645	(7.66 ± 0.55%)	NFL
	3158	(7.54 ± 0.29%)	623	(7.74 ± 0.35%)	NFM
	2872	(7.91 ± 0.62%)	589	(8.11 ± 0.63%)	NFH
	3124	(6.91 ± 0.61%)	654	(6.81 ± 0.37%)	INT
VGLUT2	2435	(6.13 ± 0.18%)	567	(6.25 ± 0.41%)	NFL
	3214	(5.84 ± 0.37%)	577	(6.11 ± 0.40%)	NFM
	3224	(6.53 ± 0.42%)	554	(6.38 ± 0.37%)	NFH
	2927	(5.63 ± 0.22%)	592	(5.71 ± 0.33%)	INT
VGAT	2742	(8.99 ± 0.65%)	587	(7.99 ± 0.37%)	NFL
	3172	(9.91 ± 0.44%)	634	(8.84 ± 0.35%)	NFM
	2852	(9.29 ± 0.44%)	656	(9.41 ± 0.42%)	NFH
	2418	(11.73 ± 0.23%)	612	(12.09 ± 0.38%)	INT
PSD-95	2841	(19.21 ± 0.45%)	623	(18.31 ± 0.72%)	NFL
	3042	(20.62 ± 0.80%)	667	(21.23 ± 0.85%)	NFM
	2776	(20.49 ± 0.66%)	621	(20.12 ± 0.75%)	NFH
	2914	(20.74 ± 0.51%)	615	(19.78 ± 0.75%)	INT
GEPH	2341	(15.92 ± 0.60%)	633	(18.04 ± 0.85%)	NFL
	3022	(18.40 ± 1.58%)	578	(18.11 ± 0.77%)	NFM
	2676	(18.80 ± 0.43%)	556	(19.07 ± 0.72%)	NFH
	2425	(19.61 ± 0.74%)	613	(20.21 ± 0.77%)	INT

*Values are mean ± SEM*.

**Table 3:** Colocalization of NFL, NFM, NFH and INT in glutamatergic and GABAergic synapses in the cerebral cortex.

	Synapse (no.)	Presynaptic compartment	Postsynaptic compartment	Both	NF subunits
VGLUT1/PSD-95	752	(14.10 ± 0.69%)	(25.05 ± 0.79%)	(3.93 ± 0.41%)	NFL
	738	(18.64 ± 0.57%)	(28.40 ± 0.41%)	(3.48 ± 0.48%)	NFM
	644	(13.49 ± 0.31%)	(23.98 ± 0.58%)	(3.33 ± 0.29%)	NFH
	781	(16.51 ± 0.74%)	(23.28 ± 0.45%)	(3.31 ± 0.42%)	INT
VGLUT2/PSD-95	756	(14.07 ± 0.53%)	(13.76 ± 0.50%)	(4.12 ± 0.63%)	NFL
	818	(16.31 ± 0.76%)	(15.30 ± 0.69%)	(3.09 ± 0.43%)	NFM
	724	(15.21 ± 0.82%)	(14.09 ± 0.97%)	(3.46 ± 0.55%)	NFH
	691	(15.60 ± 0.67%)	(15.36 ± 0.41%)	(3.27 ± 0.42%)	INT
VGAT/GEPH	750	(21.45 ± 1.00%)	(23.56 ± 1.17%)	(3.89 ± 0.26%)	NFL
	644	(29.94 ± 1.11%)	(28.21 ± 0.39%)	(3.22 ± 0.28%)	NFM
	628	(21.77 ± 0.78%)	(22.82 ± 1.91%)	(3.34 ± 0.27%)	NFH
	732	(23.53 ± 0.56%)	(24.28 ± 0.64%)	(4.07 ± 0.36%)	INT

*Values are mean ± SEM*.

NFs expression in VGLUT1+ puncta was studied in 20 sections from three rats (at least five sections/rat). The results showed that 7.11% of VGLUT1+ puncta expressed NFL, 7.54% expressed NFM, 7.91% expressed NFH and 6.91% expressed INT. Analysis of VGLUT2+ puncta (24 sections, three rats; at least five sections/rat) showed that 6.13% of VGLUT2+ puncta expressed NFL, 5.84% expressed NFM, 6.53% expressed NFH and 5.63% expressed INT. Evaluation of VGAT+ puncta (24 sections, three rats; at least five sections/rat) disclosed that 8.99% expressed NFL, 9.91% expressed NFM, 9.29% expressed NFH, and 11.73% expressed INT. As regards the postsynaptic markers, 19.21% of PSD-95+ puncta expressed NFL, 20.62% expressed NFM, 20.49% expressed NFH and 20.74% expressed INT (20 sections, three rats; at least four sections/rat). Finally, 15.92% of GEPH+ puncta expressed NFL, 18.40% expressed NFM, 18.80% expressed NFH and 19.61% expressed INT (22 sections, four rats; at least four sections/rat; Table 2 for details). The percentage of colocalization of NF proteins with markers used here did not exhibit a significantly different laminar distribution.

**Figure 5:**
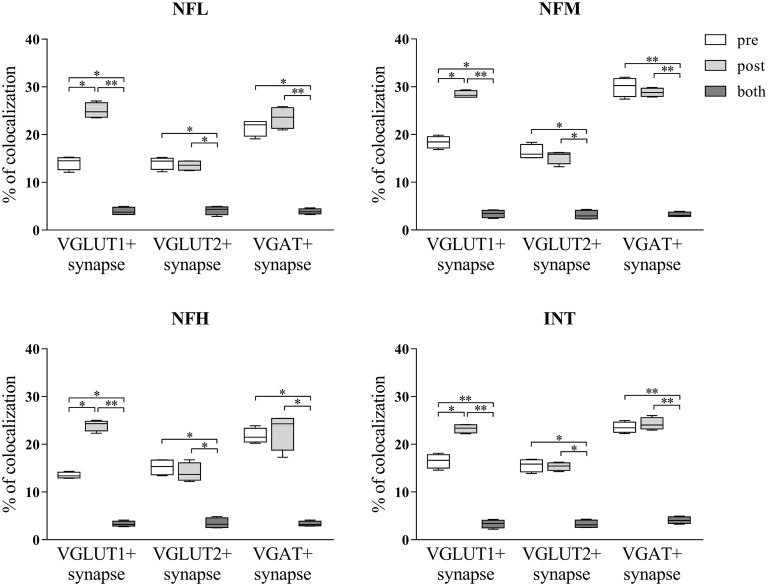
NFL, NFM, NFH and INT expression in the presynaptic compartment, postsynaptic compartment or both in glutamatergic (identified by VGLUT1+ or VGLUT2+ puncta contacting PSD-95+ puncta) and GABAergic (identified by VGAT+ puncta contacting GEPH+ puncta) synapses in the cerebral cortex. Values are mean ± SEM. Statistical significance was evaluated by non-parametric Kruskal-Wallis test with Dunn’s post-test; **p < 0.01, *p < 0.05.

Since it is possible to identify synapses in brain sections by light microscopy (Melone et al., 2005) and to differentiate presynaptic from postsynaptic sites by confocal microscopy (Bragina et al., 2015), we performed triple-labeling experiments to investigate NF expression at synapses. Glutamatergic synapses were identified by VGLUT1+ or VGLUT2+ puncta contacting PSD-95+ puncta and GABAergic synapses by VGAT+ puncta contacting GEPH+ puncta. In VGLUT1/PSD-95 synapses, 14.10% of VGLUT1+puncta expressed NFL, 18.64% expressed NFM, 13.49% expressed NFH, and 16.51% expressed INT; 25% of PSD-95+ puncta expressed NFL, 28.40% expressed NFM, 23.98% expressed NFH, and 23.28% expressed INT (Figure 5; Table 3 for details). In VGLUT2/PSD-95 synapses 14% of VGLUT2+ elements expressed NFL, 16.31% expressed NFM, 15.21% expressed NFH, and 15.60% expressed INT; 13.76% of PSD-95+ puncta expressed NFL, 15.30% expressed NFM, 14.09% expressed NFH, and 15.36% expressed INT (Figure 5; Table 3 for details). In GABAergic synapses, 21.45% of VGAT+ puncta expressed NFL, 29.94% expressed NFM, 21.77% expressed NFH, and 23.53% expressed INT; 23.56% of GEPH+ contacts expressed NFL, 28.21% expressed NFM, 22.82% expressed NFH, and 24.28% expressed INT (Figure 5; Table 3 for details). The expression of the four NF subunits were mostly detected either in the pre- or the postsynaptic compartment, and only rarely in both (Figure 5; Table 3 for details). Our data show that at VGLUT1/PSD-95 synapses NF expression is greater in the postsynaptic compartment than at presynaptic sites, in line with previous work (Yuan et al., 2015); that NF expression is higher at presynaptic sites in GABAergic terminals than at glutamatergic terminals; and that postsynaptic expression is lower at VGLUT2/PSD-95+ puncta.

## Discussion

The present findings show for the first time that NFs are localized at some cortical synapses, and that they are differentially expressed in glutamatergic and GABAergic synapses.

Synapses can be reliably identified in brain tissue by confocal microscopy, which is more appropriate than electron microscopy to examine large samples (Melone et al., 2005). Nevertheless, some *caveats* must be kept in mind. First, although presynaptic puncta adjacent to postsynaptic puncta (as seen in the present and in previous studies) in all likelihood correspond to ultrastructural synapses (i.e., synapses as identified by electron microscopy), it is likely that a proportion of ultrastructural synapses are not detected by this method. Indeed, it is conceivable that some synapses (e.g., small synapses, or synapses in which the postsynaptic marker is expressed at low levels) can escape immunocytochemical detection. Second, presynaptic proteins, postsynaptic proteins, and NFs have different optimal depths for immunodetection. Therefore, although we paid attention in pilot analyses to select a range of depth that could allow a satisfactory visualization of the three series of antigens, it is possible that we might have slightly underestimated some labeling. Third, it is conceivable that some labeling considered positive may not be associated with synapses (e.g., sites of synthesis or transport, glial cells etc.), thus overestimating it. Fourth, we used VGAT as presynaptic marker of GABAergic synapses. VGAT does transport GABA and glycine; it follows that, although glycinergic synapses are virtually absent in neocortex (Chaudhry et al., 1998; Legendre, 2001), we could have included some of them in our analysis. Moreover, the possibility that another vesicular GABA transporter or a variant of it may exist in brain, as it does in the pancreas (Suckow et al., 2006), has never been totally ruled out. These factors might slightly alter the percentages reported here, but they should not modify significantly the present results, and particularly so considering the aims of our study (see “Introduction” section).

Triple-labeling studies enabled us to analyze NF localization in a large sample of glutamatergic and GABAergic synapses. Data analysis demonstrated differential NF localization; in particular, at VGLUT1 glutamatergic synapses (recognized by VGLUT1+ puncta contacting PSD-95+ puncta) their localization was greater at postsynaptic than presynaptic sites, in line with the study by Yuan et al. (2015); whereas at VGLUT2 glutamatergic synapses (identified by VGLUT2+ puncta contacting PSD-95+ puncta) it was similar in both compartments. NF localization was also similar in pre- and postsynaptic elements of GABAergic synapses (identified by VGAT contacting GEPH+ puncta). Moreover, a similar presynaptic NF localization was detected in the two types of glutamatergic synapses. Presynaptic NF localization was greater at GABAergic than glutamatergic synapses, whereas in the postsynaptic compartment it was comparable in GABAergic and VGLUT1+ glutamatergic synapses (but lower in VGLUT2+ glutamatergic synapses). Our data showed a lower NF expression in VGLUT1+, VGLUT2+ and VGAT+ puncta compared with VGLUT1+, VGLUT2+ and VGAT+ puncta contacting PSD-95+ or GEPH+ puncta. In other words, NF expression was greater in the presynaptic compartment if the presynaptic elements contacted postsynaptic structures; the same was true of postsynaptic NF expression, although the difference was less marked. We first ascribed this finding to methodological variability due to the antibodies, since we used monoclonal antibodies in double-labeling experiments and polyclonal antibodies in triple-labeled sections. However, calculation of the percentage of colocalization of the vesicular transporters with NFs in triple-labeled sections with the same method used for double-labeling studies (i.e., including all puncta) gave similar results, implying that the use of different antibodies is not responsible for this observation. Although the influence of other factors (e.g., an extrasynaptic localization of the proteins) cannot be ruled out, it is reasonable to conclude that presynaptic NF expression is lower at synapses lacking the postsynaptic compartment or postsynaptic proteins. Indeed, excitatory and inhibitory spine synapses in the adult cerebral cortex are extremely plastic (Knott et al., 2006; Chen et al., 2012; Villa et al., 2016), and a large fraction of newly formed spines does not immediately express PSD-95, whereas some spines that eventually disappeared lack PSD-95 (Cane et al., 2014). Therefore, the higher NF expression found in presynaptic elements contacting postsynaptic structure may reflect a greater NF expression at functional synapses.

NFs have been shown to play a functional role at synapses, since their elimination disrupts synaptic plasticity and impairs social memory, with severe implications for some neuropsychiatric diseases (Yuan et al., 2015). In different cerebral structures and neurotransmitter systems the mechanism seems to be related to receptor anchoring or localization in the neuronal plasma membrane, at least at postsynaptic sites; indeed, the lack of NFM leads to changes in D1 receptor-mediated LTP and behavior, while NFL is capable to directly bind and anchor NMDA receptors (Ehlers et al., 1998; Ratnam and Teichberg, 2005; Yuan et al., 2015). Since synaptic strength can be regulated by controlling the abundance of neurotransmitter receptors at synapses through targeted insertion and removal (Craig, 1998), NFs may be involved in synaptic plasticity through an action on receptor expression. NFs action on receptors could not be limited to this, however, as it may also include changes in the stoichiometry of NFs subunits (Chinnakkaruppan et al., 2009). Present knowledge prevents a clearer hypothesis on the role of NFs in synaptic function and plasticity, and it is clear that much work is needed to increase our understanding of these proteins in synaptic physiology. However, by demonstrating that NFs are expressed at cortical synapses in a synapse subtype-differential manner, the present results may pave the way to future studies that will unravel the molecular mechanisms of NFs at synapses and their alterations in different physiological and pathophysiological conditions, e.g., in the mechanisms of neural plasticity and of neurodegenerative diseases characterized by intraneuronal accumulation of NFs, like Alzheimer’s and Parkinson’s diseases (Vickers et al., 2016; Yuan and Nixon, 2016; Yuan et al., 2017).

## Author Contributions

FC conceived the project. LB performed the experiments and gathered and analyzed the data. FC supervised the project and discussed the data. LB and FC wrote the article.

## Conflict of Interest Statement

The authors declare that the research was conducted in the absence of any commercial or financial relationships that could be construed as a potential conflict of interest.
